# Promoting Physical Activity and Weight Loss With mHealth Interventions Among Workers: Systematic Review and Meta-analysis of Randomized Controlled Trials

**DOI:** 10.2196/30682

**Published:** 2022-01-21

**Authors:** Jiyeon Jung, Inhae Cho

**Affiliations:** 1 Department of Nursing Korea National Open University Seoul Republic of Korea; 2 College of Nursing Korea University Seoul Republic of Korea

**Keywords:** mHealth, physical activity, obesity, weight loss, workforce, workplace health promotion, mobile phone

## Abstract

**Background:**

Physical activity (PA) is a vital factor in promoting health in the workforce. Mobile health (mHealth) interventions have recently emerged in workplace health promotion as an effective strategy for inducing changes in health behaviors among workers; however, the effectiveness of mHealth interventions in promoting PA and weight loss for workers is unclear.

**Objective:**

This study aims to provide a comprehensive analysis of current evidence on the effectiveness of mHealth interventions in promoting PA and weight loss among workers.

**Methods:**

We searched relevant databases, including PubMed, Embase, CINAHL Complete, and the Cochrane Library, for publications on mHealth interventions in the English or Korean language from inception to December 2020. Randomized controlled trials that evaluated the effectiveness of mHealth in improving PA and weight loss were retrieved. A meta-analysis with a random effects model and subgroup analyses was performed on PA types and mHealth intervention characteristics.

**Results:**

A total of 8 studies were included in this analysis. More than half of the studies (5/8, 63%) were identified as having a high risk of bias. The mHealth intervention group showed a significant improvement in PA (standardized mean difference [SMD] 0.22, 95% CI 0.03-0.41; *P*<.001; *I*^2^=78%). No significant difference in weight loss was observed when comparing the intervention group with the control groups (SMD 0.02, 95% CI –0.07 to 0.10; *P*=.48; *I*^2^=0%). A subgroup analysis was also performed; walking activity (SMD 0.70, 95% CI 0.21-1.19; *P*<.001; *I*^2^=83.3%), a multicomponent program (SMD 0.19, 95% CI 0.05-0.33; *P*=.03; *I*^2^=57.4%), objective measurement (SMD 0.58, 95% CI 0.05-1.10; *P*<.001; *I*^2^=87.3%), and 2 or more delivery modes (SMD 0.44, 95% CI 0.01-0.87; *P*<.001; *I*^2^=85.1%) were significantly associated with an enhancement in PA.

**Conclusions:**

This study suggests that mHealth interventions are effective for improving PA among workers. Future studies that assess long-term efficacy with a larger population are recommended.

## Introduction

### Background

The promotion of physical activity (PA) is reported to be a key strategy for health promotion. Regular PA is proven to help prevent and treat noncommunicable diseases, such as cancer, cardiovascular disease, diabetes, stroke [1], and cardiovascular disease mortality [2]. It can also prevent hypertension [3] and obesity [4] and improve health-related quality of life [5].

According to the World Health Organization, 25% of adults do not currently meet the PA recommendations [6]. Thus, the World Health Organization provides a global action plan and framework for practical and feasible policy actions to support, maintain, and increase PA [6]. Establishing and maintaining healthy lifestyles in the adult population is essential [7], and it should be noted that most of the adult population are workers [8]. Inadequate PA is identified as a significant problem in adult worker groups [9,10]. This is mainly owing to the decrease in the amount of nonwork activity of blue-collar workers and white-collar workers who have sedentary behavior during work [11].

Most employed adults spend a large part of their waking hours at work [12]; thus, workplaces provide a unique and fruitful health promotion setting that can significantly increase PA and potentially influence workers’ health [13]. In addition, promoting workers’ PA was reported to be potentially beneficial, improving health status and psychological well-being and increasing economic benefits for employers through increased productivity [14,15]. However, there are several barriers to PA, of which one of the most widely mentioned is a lack of time [15].

Improving PA through mobile technology (mobile health [mHealth]) is emerging as a major trend in workplace health promotion for interventional change [16]. mHealth is based on wireless devices and sensors that people wear during their daily activities, including mobile phones and is reported to be convenient and effective in changing health behavior [17,18]. In particular, it is recognized as a tool for intervention delivery that enables continuous monitoring during daily life and various interventions [10], thus enhancing one’s responsibility for their own health and performance [19]. The proper use of mobile technologies for promoting PA may be a cost-effective and feasible way to reach this population [20].

Previous studies have investigated the use of mHealth to promote PA in various populations, including workers [21-31]. A study on mHealth apps and self-determination theory showed increased PA levels in motivated workers [30]. In addition, a large population-based mHealth intervention study reported significant improvements in PA, sitting times, and body weight [31]. mHealth devices not only track data but also encourage workers to achieve their health goals through sustained engagement [32]. A previous review concluded that mHealth interventions are potentially effective and feasible for increasing PA in the workplace [33], with some evidence of short-term weight loss [34]. In contrast, other studies reported nonsignificant changes in PA level [35] and weight control [7,24] in certain groups of workers.

There is a knowledge gap on the effectiveness of mHealth technologies in promoting PA [30] and weight loss [24] among workers. Furthermore, findings from the current literature are still inconclusive [33]. There is still some debate about the effectiveness of mHealth interventions in the working population.

### Objective

In this study, we aim to provide a comprehensive analysis of current evidence from randomized controlled trials (RCTs) on the effectiveness of mHealth interventions in promoting PA and weight loss among workers.

## Methods

### Study Design

This study is a systematic review and meta-analysis of RCTs conducted according to the PRISMA (Preferred Reporting Items for Systematic Reviews and Meta-Analyses) guidelines [36].

### Search Strategy

A literature review of 4 bibliographic electronic databases—PubMed, Embase, Cochrane Library, and CINAHL Complete—was conducted. Published articles on mHealth from its inception until December 2020 were identified. We confirmed the search terms based on our research question. According to search terms, the Medical Subject Headings terms; Emtree, the related entry term; and free terms were collected from relevant articles and bibliographic databases. The keywords identified were as follows: *telemedicine*, *cell phone*, *smartphone*, *mobile device*, *mHealth*, *mobile applications*, *mHealth program*, *worker*, *employee*, *occupation worksite*, *working adult*, *workplace*, *occupational health*, *randomized controlled trial*, *clinical trial*, *controlled clinical trial*, *evaluation study*, and *quasi-experimental*. Our search strategies are presented in Multimedia Appendix 1.

After the search, relevant identified articles were exported using the bibliography software Endnote (Version X9.1; Clarivate Analytics) and duplicate papers were removed. The titles and abstracts were screened by 2 reviewers (JJ and IC) independently, using preset criteria; irrelevant publications were excluded and full-text articles were then selected. To identify additional studies, we manually checked the reference lists of relevant reviews found in the original search. The entire process, from developing a search strategy to selecting studies and cross-checking all publications, was carried out by the 2 reviewers (JJ and IC). In cases of inconsistent selection, an agreement was reached through discussion.

### Inclusion and Exclusion Criteria

This study’s eligibility criteria were specified according to the purpose of this review. On the basis of the participants, intervention, comparison, outcome, and study design framework, the inclusion criteria were as follows: (1) participants, working population and those aged ≥18 years; (2) intervention, any mHealth intervention that promoted PA using mobile technologies (mHealth interventions were programs that used mobile phones with mobile functions, such as phone call, message service, app, GPS, Bluetooth technology, and others); (3) comparison, control group should refer to participants who did not receive any intervention using mobile phones; (4) outcome, the study’s outcome included PA (eg, self-reported or device-reported PA, walking time, and the number of steps) or body weight to verify the effects of mHealth interventions on promoting PA and weight reduction in workers; and (5) study design, only the RCT design was considered.

The study’s exclusion criteria included the following: (1) studies not published in English or Korean, (2) studies that targeted participants with a disease, (3) studies that reported incomplete or insufficient data (eg, study protocols, ongoing studies, and conference abstract), and (4) studies with web-based mHealth intervention.

### Data Extraction

The data from the eligible studies were extracted using Excel (Microsoft Corporation) and were coded using a predesigned template by the research team. The data included general study characteristics (eg, author, published year, country, setting, design, participants, their age, and comparator), intervention characteristics (eg, mHealth intervention delivery mode, category, intervention contents, behavior change techniques, and duration), and the study’s result (eg, outcome variables).

It has been reported that mHealth interventions are often performed together with various intervention components in workers’ health promotion programs [33]. Thus, we classified intervention into 2 different categories. The included studies were classified into a stand-alone mHealth intervention using mobile device only or a multicomponent intervention where the use of mHealth device was one of several intervention components in the programs (eg, face-to-face counseling, printed materials, offline education, and organizational support). Finally, for the coding of behavior change techniques, we used the Coventry, Aberdeen, and London-Refined taxonomy by Michie et al [37]. This 40-item taxonomy can be used to systematically classify PA and healthy eating behaviors.

Data extraction was performed independently by the first reviewer (JJ) and confirmed by the second reviewer (IC). When discrepancies emerged, we resolved them through discussion until an agreement was reached.

### Risk of Bias Assessment

The eligible studies were evaluated for the risk of bias using a revised Cochrane risk of bias tool. This tool was developed to assess the risk of bias in randomized trials [38]. The Cochrane risk of bias tool consists of the following five domains: (1) randomization process, (2) deviations from intended interventions, (3) missing outcome data, (4) measurement of the outcome, and (5) selection of the results. The risk of bias was evaluated using algorithms that depend on the answers to the questions in each domain. As a result, each domain was assigned 1 of 3 levels (*high risk*, *low risk*, and *some concerns*). The risk of bias in the included studies was assessed by combining the results across the domain responses. The 2 reviewers (JJ and IC) independently assessed the risk of bias in each article. If there were differences in evaluation between the 2 reviewers, they were discussed and resolved.

### Statistical Analysis

The extracted data from the included studies were analyzed using Stata 17.0 (StataCorp LLC). We used a random effects model in this analysis. A meta-analysis was performed using continuous data. The standardized mean difference (SMD) was calculated as the Hedges *g* using mean and SDs. For extracted data without mean and SD, the Hedges *g* was estimated using other statistical data (eg, mean difference [MD], *P* value, and CI) using Comprehensive Meta-Analysis version 3 (Biostat Inc). The heterogeneity within selective studies was estimated using the statistic *I*^2^ [39]. Subgroup analysis was conducted according to PA features, intervention category, the PA measurement, and the number of delivery modes of the mHealth program.

## Results

### Search Results

**Figure 1:**
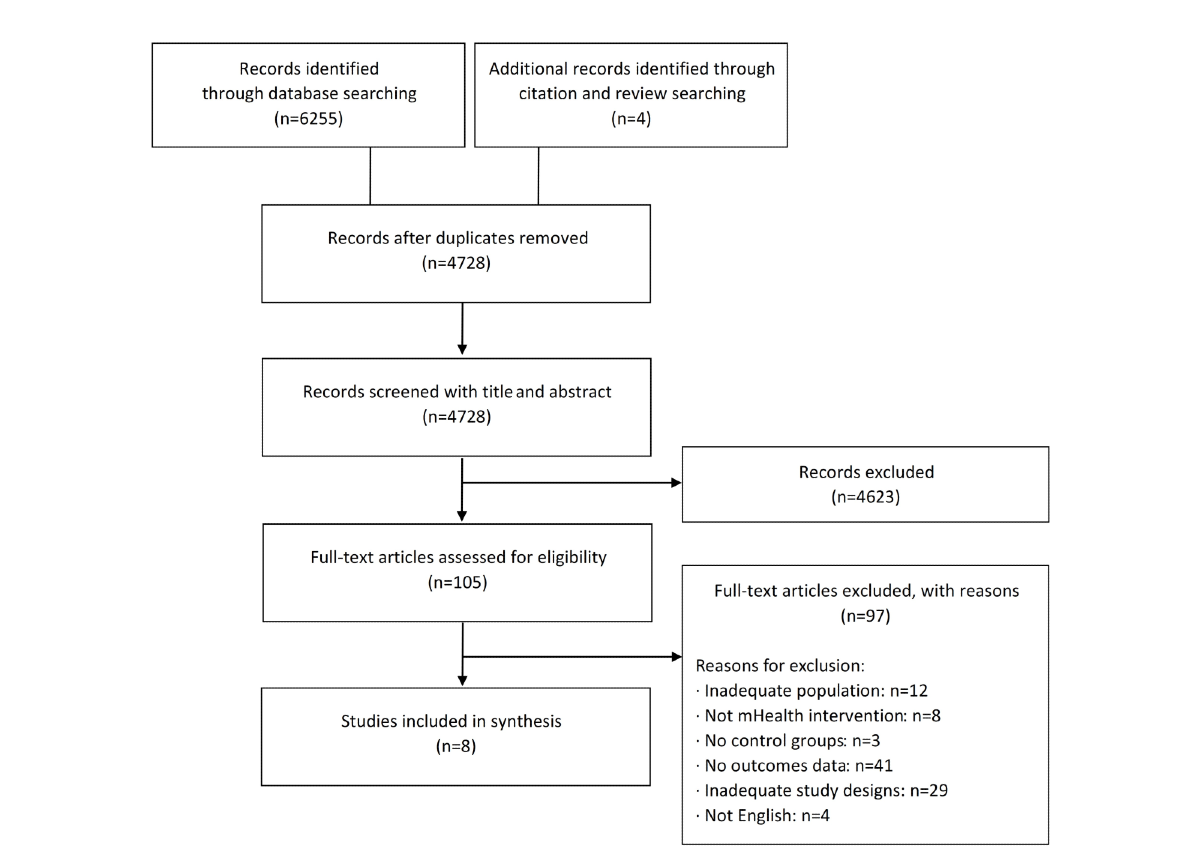
Flow diagram of study selection. mHealth: mobile health.

The search identified 6255 records in the bibliographic databases, and an additional 4 records were added through manual search from relevant reviews. After excluding duplicate records, the study titles and abstracts were screened; of the 6259 studies, 4623 (73.86%) studies that did not meet the eligibility criteria were excluded and the remaining 105 (1.67%) studies were checked. After a full-text review, 0.13% (8/6259) of the studies met the study eligibility criteria and were included in the meta-analysis (Figure 1) [22-29].

### Risk of Bias

**Figure 2:**
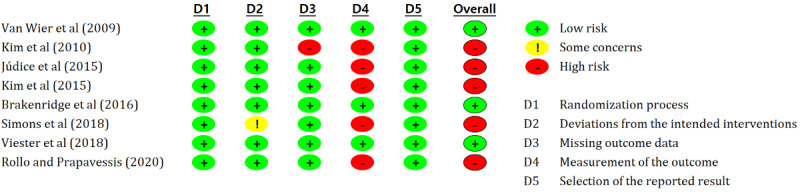
Results of risk of bias assessment for the included studies using Cochrane risk of bias tool 2.0 (detailed assessment of included studies) [22-29].

**Figure 3:**
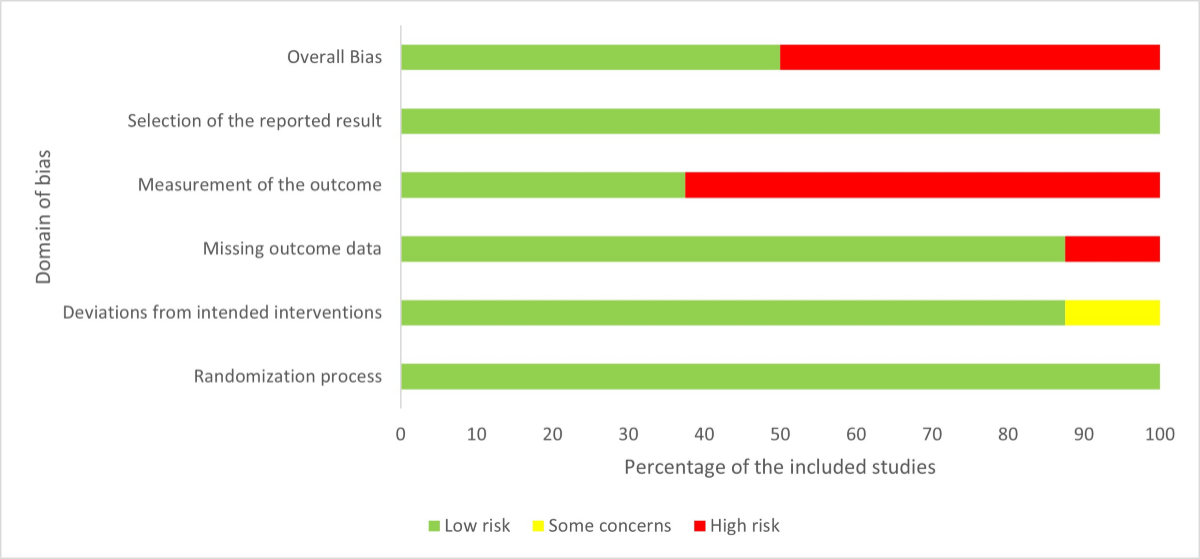
Results of risk of bias assessment for included studies using Cochrane risk of bias tool 2.0.

More than half of the studies (5/8, 63%) had a high risk of bias [23-25,27,29]. The risk of bias in the measurement of outcome was considered high in 63% (5/8) of the studies owing to self-report methods without sufficient blinding [23-25,27,29]. In the study by Kim et al [25], there was a high risk of bias for missing outcome data. All 8 studies had a low risk of bias in the randomization process and in selecting the reported result (Figures 2 and 3).

### Study Characteristics

These meta-analysis studies were published between 2009 and 2020. Among the 8 studies, there were 2 (25%) studies each from the Netherlands and the United States and 1 (13%) study each from Canada, Portugal, Korea, and Belgium. Most studies had a 2-arm RCT design (5/8, 63%); the rest had a 3-arm RCT (1/8, 13%), cluster RCT (1/8, 13%), and crossover RCT (1/8, 13%). Most participants were healthy workers; only 13% (1/8) of the studies targeted obese employees (Table 1). Half of the included studies (4/8, 50%) focused only on PA, and the other studies (4/8, 50%) focused on the contents of PA and dietary change (Multimedia Appendix 2) [22-29].

**Table 1:** Characteristics of the included studies (N=8).

Study	Country	Setting	Design	Participants	Age (years), mean (SD)	Comparator	Outcomes	
							PA^a^ (measurement)	BW^b^ or BMI
van Wier et al [28]	Netherlands	7 companies (IT^c^, hospitals, insurance, bank, and police force)	3-arm RCT^d^; IG^e^1: phone group; IG2: email group; CG^f^: control group	1386^g^; 929 (67.02%) male participants, 457 (32.97%) female participants; IG1 (n=462, 33.33%); IG2 (n=464, 33.47%); CG (n=460, 33.19%)	IG1: 43 (8.8); IG2: 43 (8.4); CG: 43 (8.7)	Printed materials	MET^h^ minutes per week (SR^i^: SQUASH^j^)	BW
Kim et al [25]	United States	43 companies and 13 community organizations	2-arm RCT	2470; IG (n=1279, 51.78%): 238 (18.6%) male participants, 1041 (81.39%) female participants; CG (n=1191, 48.22%): 246 (20.73%) male participants, 945 (79.34%) female participants	IG: 43.5 (10.3); CG: 43.6 (10.1)	Printed materials	Time of mild, moderate, and vigorous PA (SR: minutes per day)	BW
Júdice et al [23]	Portugal	Academic and administrative sectors of the university and others	Crossover RCT	10; 5 (50%) male participants, 5 (50%) female participants; IG (n=5, 50%); CG (n=5, 50%)	50.4 (11.5)	Usual care	Time of sitting and standing, walking time (hours per day), number of steps, and sitting time (OB^k^: ActivPAL)	N/A^l^
Kim et al [24]	Korea	3 public institutions	2-arm RCT	205 (n=196, 95.6% for analysis); 100% (196/196) male participants; IG (n=101, 51.5%); CG (n=95, 48.5%)	IG: 41.02 (6.82); CG: 41.55 (6.98)	Printed materials and face-to-face counseling	MET minutes per week (SR: IPAQ^m^)	BW
Brakenridge et al [22]	United States	An international property and infrastructure company	Cluster RCT	153; 83 (54.2%) male participants, 70 (45.8%) female participants; IG (n=66, 43.1%); CG (n=87, 56.9%)	IG: 37.6 (7.8); CG: 40 (8)	Organizational support	Time of sitting and standing, walking time, and number of steps (OB: ActivPAL)	N/A
Simons et al [27]	Belgium	29 workplaces (shops, retail stores, catering industry, social employment businesses, factories, etc)	2-arm RCT	130; 63 (48.5%) male participants, 67 (51.5%) female participants; IG (n=60, 46.2%); CG (n=70, 53.8%)	IG: 24.8 (3.1); CG: 25.1 (3)	Printed materials	Time of light, moderate, and vigorous PA; MVPA^n^ and total PA (OB: GT3X and accelerometers); occupational, household, recreational, active transport, and total PA (SR: IPAQ), and number of steps (OB: Fitbit Charge)	BMI
Viester et al [29]	Netherlands	A construction company	2-arm RCT	314; 100% (314/314) male participants; IG (n=162, 51.6%); CG (n=152, 48.4%)	IG: 46.3 (9.9); CG: 47 (9.5)	Usual care	Time of leisure-time MVPA (SR: SQUASH)	BW
Rollo and Prapavessis [26]	Canada	Large businesses, office spaces, and universities	2-arm RCT	60; 5 (8%) male participants, 55 (92%) female participants; IG (n=29, 48%); CG (n=31, 52%)	IG: 46.59 (11.13); CG: 43.87 (11.54)	Usual care	Time of sitting and standing, walking time, and stretching (SR: OSPAQ^o^)	N/A

^a^PA: physical activity.

^b^BW: body weight.

^c^IT: information technology.

^d^RCT: randomized controlled trial.

^e^IG: intervention group.

^f^CG: control group.

^g^Included overweight employees.

^h^MET: metabolic equivalent task.

^i^SR: self-reported.

^j^SQUASH: Short Questionnaire to Assess Health-Enhancing Physical Activity.

^k^OB: objective.

^l^N/A: not applicable.

^m^IPAQ: International Physical Activity Questionnaire.

^n^MVPA: moderate to vigorous physical activity.

^o^OSPAQ: Occupational Sitting and Physical Activity Questionnaire.

### The mHealth Intervention

We identified the program characteristics of the included studies to confirm the features of mHealth interventions on PA among workers (Table 2). We classified them as follows: mHealth intervention delivery mode, intervention category, mHealth intervention contents, behavior change techniques, PA features, and duration (with or without follow-up).

The mHealth intervention delivery mode included phone calls, SMS text messages, wearable activity monitors, and smartphone apps. Half of the studies (4/8, 50%) included phone calls to motivate the participant to be physically active [22-24,28]; then, they were in the order of wearable activity monitors (3/8, 38%) [24,26,27], SMS text messages (3/8, 38%) [24,25,29], and apps (3/8, 38%) [25-27]. Half of the interventions (4/8, 50%) were implemented using 2 or more modes of delivery [24-27]. The intervention category was classified into multicomponent (6/8, 75%) [22,23,25,26,28,29] and stand-alone (2/8, 25%) [24,27]. The most used component in the multicomponent intervention was educational materials (5/8, 63%) [22,25,26,28,29]. The PA features were categorized into overall PA and walking activity. Of the 8 included studies, 4 (50%) studies reported overall PA [24,25,28,29], 3 (38%) studies reported walking activity [22,23,26], and 1 (13%) study dealt with both measurements [27]. The intervention duration ranged from 1 week to 12 months. Most studies spanned 12 months; only 3 (38%) studies reported a follow-up.

**Table 2:** Characteristics of mobile health interventions in the included studies (N=8).

Study	Delivery mode	Category	Mobile health intervention contents	Behavior change techniques	PA^a^ features	Duration (follow-up)
van Wier et al [28]	Phone call	MC^b^: face-to-face counseling and educational materials	Phone call counseling, face-to-face counseling, and printed materials	Prompt self-monitoring, provide feedback, provide instruction, teach to use prompts, goal-setting, and provide information (printed materials)	Overall PA	6 months (no follow-up)
Kim et al [25]	Phone call	MC: educational materials	Phone call counseling and printed materials	Provide information (printed materials), goal-setting, action planning, problem solving, set graded tasks, prompt review, provide feedback, provide instruction, and stress management	Overall PA	6 months (no follow-up)
Júdice et al [23]	Phone call, SMS text message, and wearable activity monitor	SA^c^	Activity monitor, alert, and feedback	Goal-setting, prompt self-monitoring, teach to use prompts, and provide feedback	Walking activity	1 week (no follow-up)
Kim et al [24]	SMS text message	MC: offline education and face-to-face counseling	Tailored SMS text message, offline education, and face-to-face counseling	Goal-setting, problem solving, prompt self-monitoring, provide feedback, provide information, provide instruction, provide information (printed materials and face-to-face counseling), and use of follow-up prompts	Overall PA	6 months (no follow-up)
Brakenridge et al [22]	Wearable activity monitor and app	MC: organizational support (emails and educational materials)	Activity monitor, feedback, and organizational support	Prompt self-monitoring, provide feedback, plan social support, and provide information (printed materials)	Walking activity	12 months (no follow-up)
Simons et al [27]	Wearable activity monitor and app	SA	Activity monitor and feedback	Goal-setting, action planning, problem solving, set graded tasks, prompt review of behavioral goals, provide information, provide feedback, and prompt self-monitoring	Overall PA and walking activity	9 weeks (12 weeks)
Viester et al [29]	Phone call	MC: educational materials and organizational support	Phone call counseling, printed materials, and organizational support	Goal-setting, problem solving, prompt review of behavioral goals, provide information, provide feedback, prompt self-monitoring, and plan social support	Overall PA	6 months (12 months)
Rollo and Prapavessis [26]	SMS text message	MC: face-to-face counseling and educational materials	Tailored SMS text message, face-to-face counseling, and printed materials	Counseling, goal-setting, action planning, problem solving, set graded tasks, provide information, and teach to use prompts	Walking activity	6 weeks (8 weeks)

^a^PA: physical activity.

^b^MC: multicomponent.

^c^SA: stand-alone.

### The Effects on PA and Weight Loss

All the 8 studies reported on PA [22-29]. Júdice et al [23], Brakenridge et al [22], and Simons et al [27] used more than 2 measurements as outcome variables; the results were included in the meta-analysis. These results were treated individually in the meta-analysis; therefore, 12 effects were analyzed in this PA meta-analysis. Results showed that the mHealth intervention group was significantly associated with an improvement in PA after completing the intervention compared with the control group (SMD 0.22, 95% CI 0.03-0.41; *P*<.001; *I*^2^=78%).

**Figure 4:**
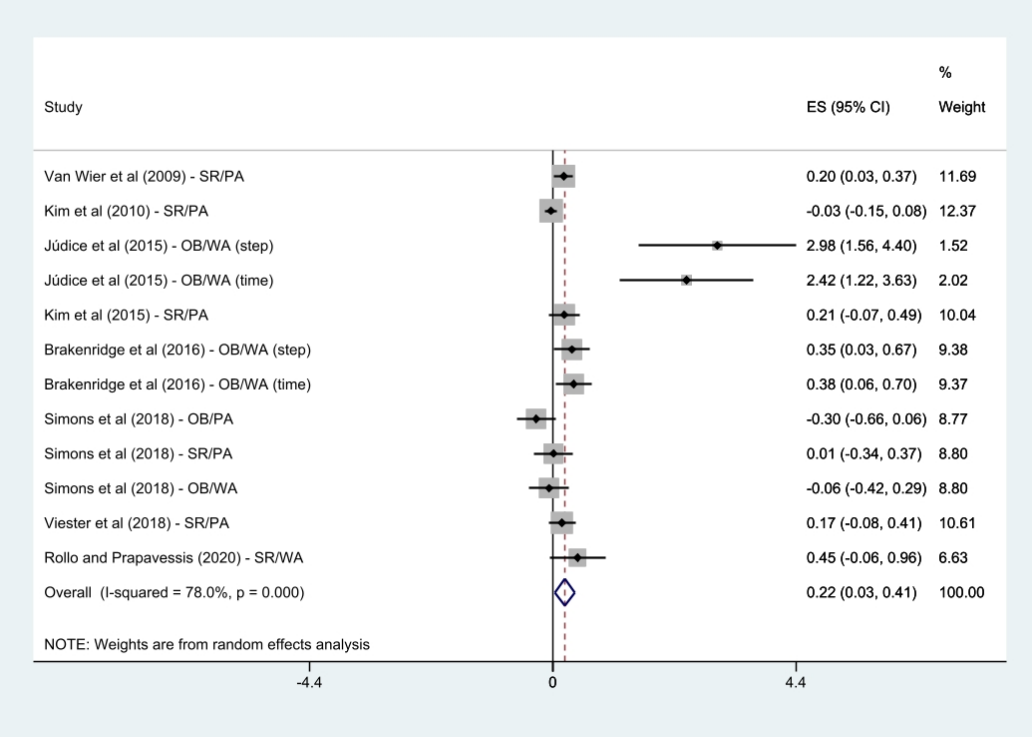
Meta-analysis of mobile health intervention effect on physical activity [22-29]. ES: effect size; OB: objective; PA: physical activity; SR: self-reported; WA: walking activity.

**Figure 5:**
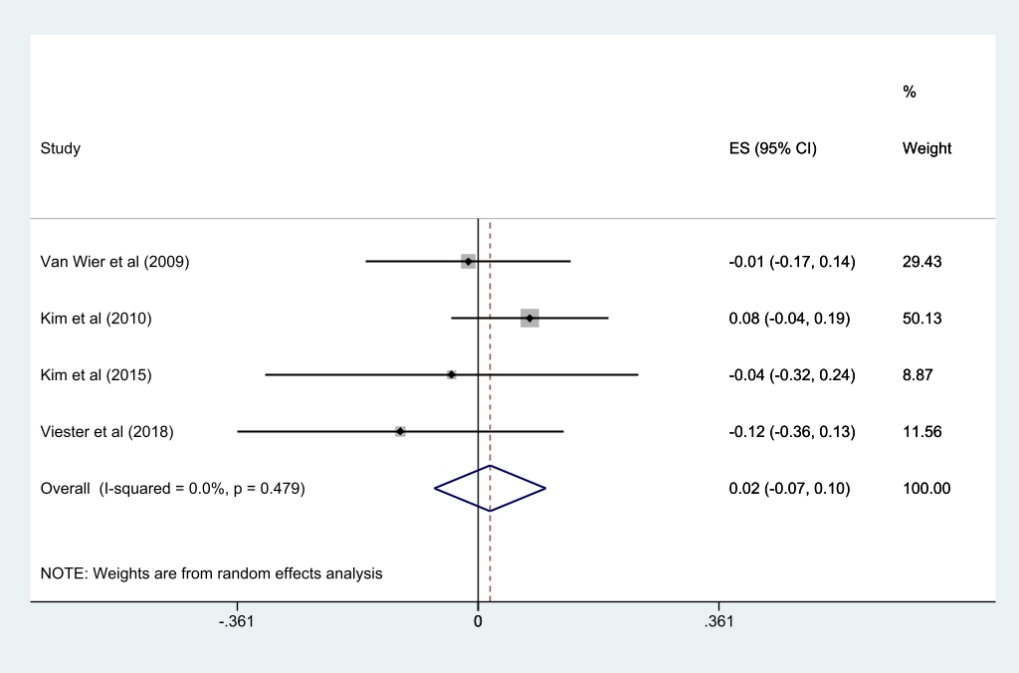
Meta-analysis of mobile health intervention effect on weight loss [24,25,28,29]. ES: effect size.

Regarding weight loss in workers, 50% (4/8) of the studies, except the study by Simons et al [27] that did not report the results of body weight, were analyzed in the meta-analysis. There was no statistically significant difference in weight loss compared with control groups (SMD 0.02, 95% CI –0.07 to 0.10; *P*=.48; *I*^2^=0%). A summary of the detailed findings is presented in Figures 4 and 5.

### Subgroup Analysis

A subgroup analysis was conducted according to PA features (overall PA or walking activity), intervention categories (multicomponent program or stand-alone mHealth program), PA measurements (self-reported measurement or objective measurement), and the number of delivery modes (1, 2, or more).

**Figure 6:**
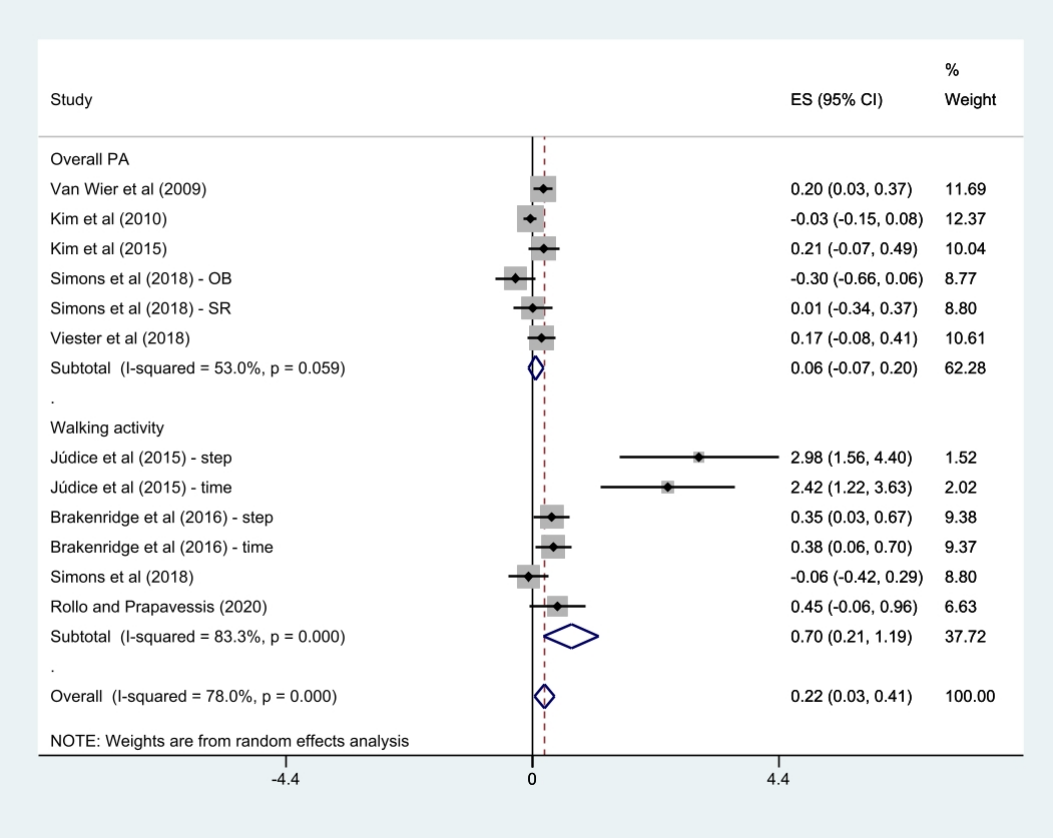
Subgroup analysis by physical activity features [22-29]. ES: effect size; OB: objective; PA: physical activity; SR: self-reported.

**Figure 7:**
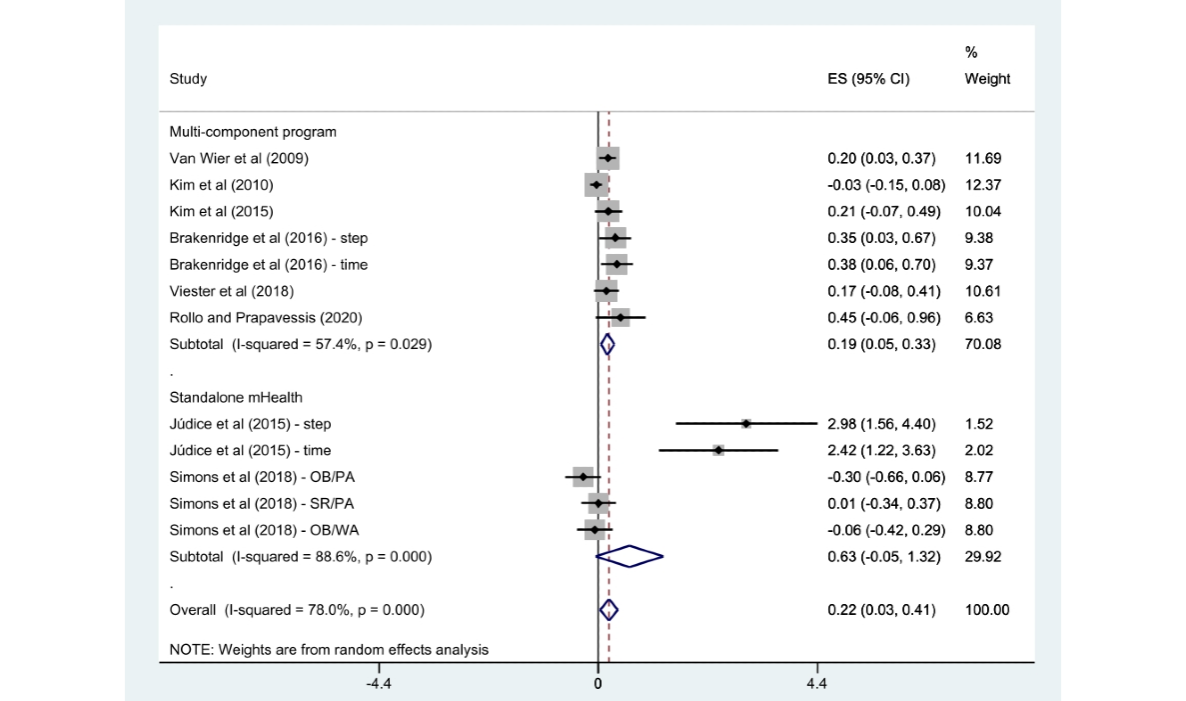
Subgroup analysis by intervention category [22-29]. ES: effect size; OB: objective; PA: physical activity; SR: self-reported; WA: walking activity.

**Figure 8:**
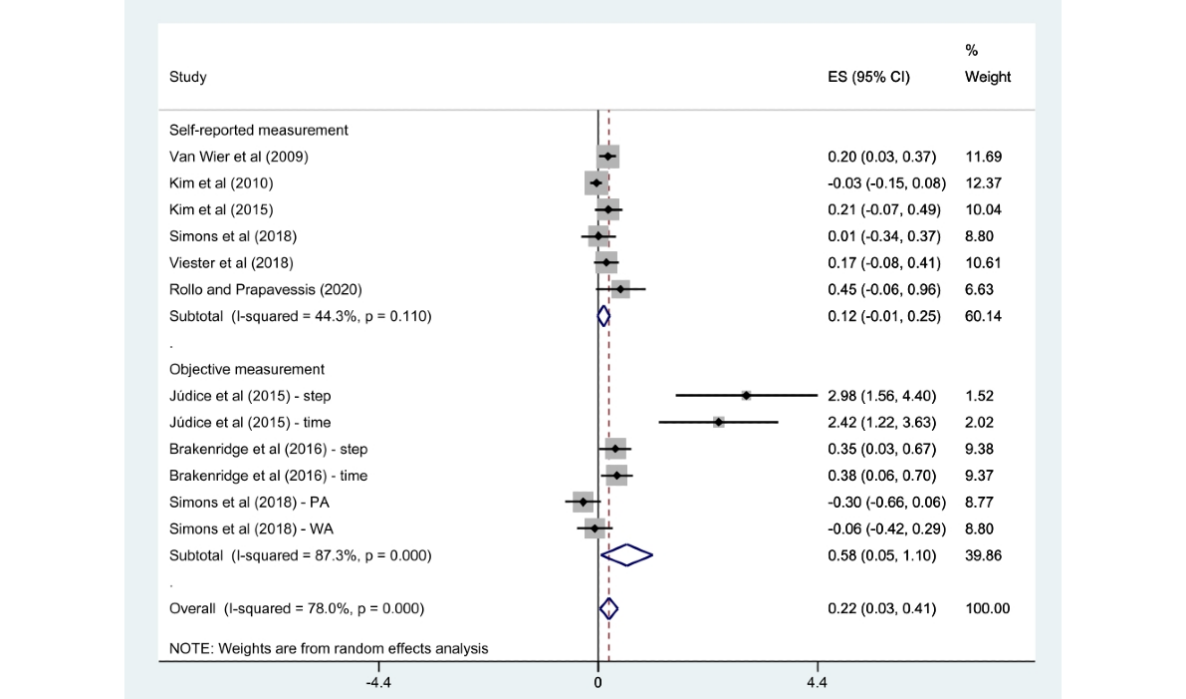
Subgroup analysis by physical activity measurements [22-29]. ES: effect size; PA: physical activity; WA: walking activity.

**Figure 9:**
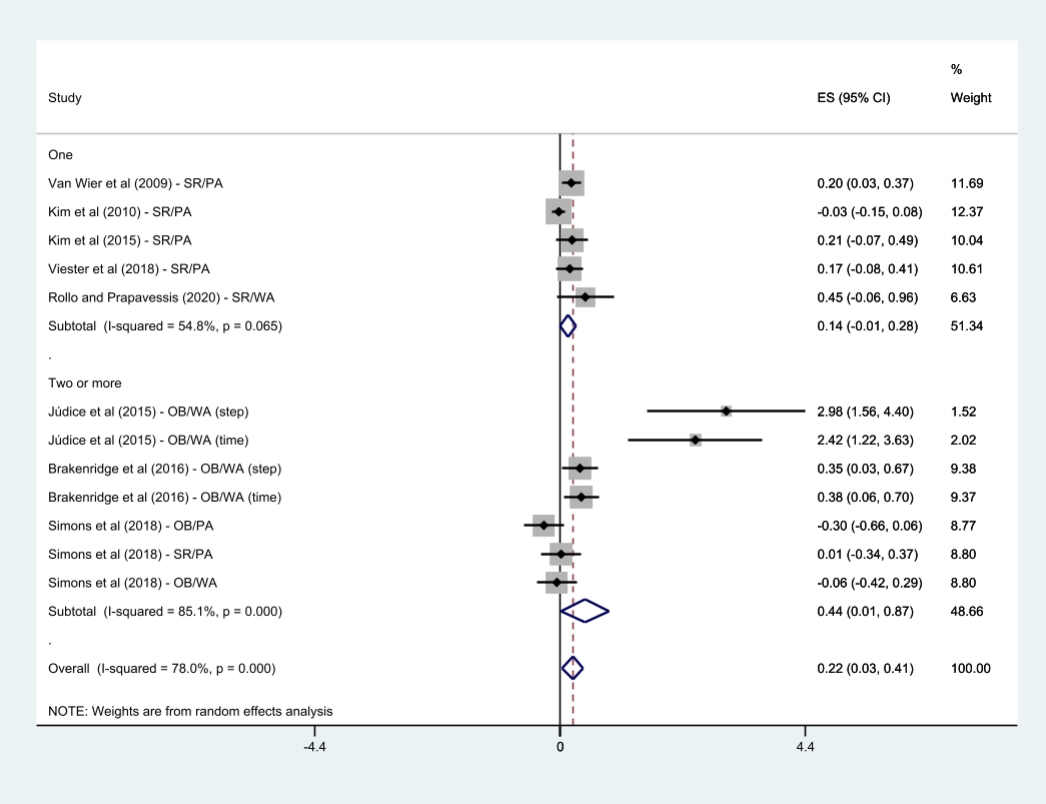
Subgroup analysis by the number of delivery modes [22-29]. ES: effect size; OB: objective; PA: physical activity; SR: self-reported; WA: walking activity.

The subgroups of walking activity (SMD 0.70, 95% CI 0.21-1.19; *P*<.001; *I*^2^=83.3%), multicomponent program (SMD 0.19, 95% CI 0.05-0.33; *P*=.03; *I*^2^=57.4%), objective measurement (SMD 0.58, 95% CI 0.05-1.10; *P*<.001; *I*^2^=87.3%), and 2 or more delivery modes (SMD 0.44, 95% CI 0.01-0.87; *P*<.001; *I*^2^=85.1%) showed a significant association with an enhancement in PA when compared with the control group. However, the overall PA (SMD 0.06, 95% CI –0.07 to 0.20; *P*=.06; *I*^2^=53%), stand-alone mHealth program (SMD 0.63, 95% CI –0.05 to 1.32; *P*<.001; *I*^2^=88.6%), self-reported measurement (SMD 0.12, 95% CI –0.01 to 0.25; *P*=.11; *I*^2^=44.3%), and 1 delivery mode (SMD 0.14, 95% CI –0.01 to 0.28; *P*=.07; *I*^2^=54.8%) demonstrated no statistically significant difference compared with the control groups. Detailed findings are presented in Figures 6-9.

### Publication Bias and Sensitivity Analysis

**Figure 10:**
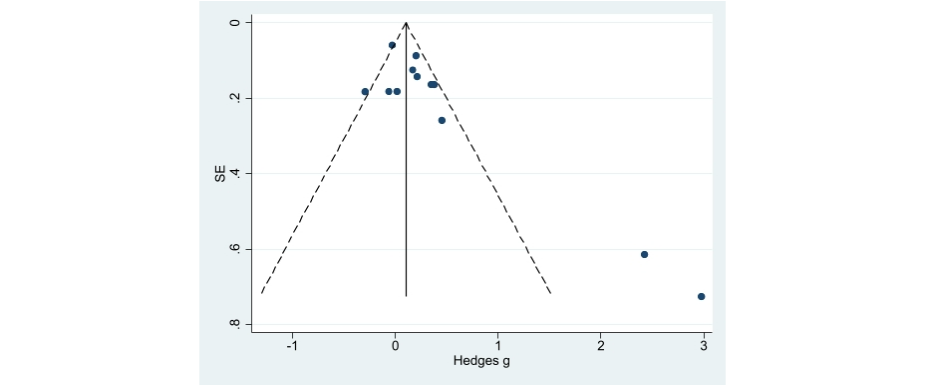
Funnel plot for publication bias assessment (physical activity).

**Figure 11:**
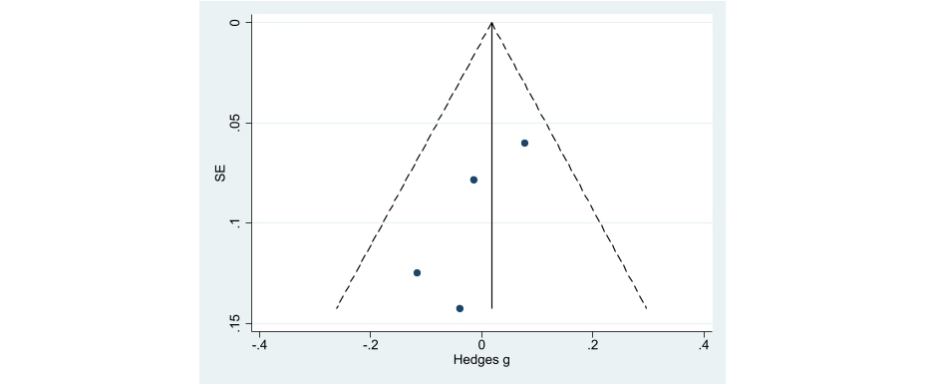
Funnel plot for publication bias assessment (weight loss).

Publication bias was assessed using a funnel plot (Figures 10 and 11). Although the funnel plot was shown to be visually asymmetrical for PA, the Begg correlation test (*P*=.15) was not statistically significant.

We conducted a sensitivity analysis to estimate the robustness of our findings (Multimedia Appendices 3 and 4). We identified the weights of the included studies and then eliminated them one by one to assess the impact of the study on the overall effects. When the study by Kim et al [25] was excluded, there was a change in the MD because its weight was the largest in PA analysis (SMD 0.19, 95% CI 0.10-0.29). After removal of other studies, the MD ranged from 0.08 to 0.19 and was similar to SMD 0.10 (95% CI 0.03-0.18) from the original value calculated using a fixed model.

## Discussion

### Principal Findings

This meta-analysis, which included only RCTs, attempted to analyze the effectiveness of mHealth interventions in PA improvement and weight loss among workers. Subgroup analysis was based on differences in PA features, intervention categories, PA measurement, and the number of delivery modes. Overall, a small to moderate effect was found in mHealth interventions for workers in PA improvement and no statistically significant difference was found in weight loss.

PA improvement was particularly observed in the walking activity feature but not for weight loss in the subgroup analysis. The results also indicated positive effects for multicomponent programs rather than stand-alone mHealth programs in improving overall PA among workers. Moreover, the objective measurement of PA and 2 or more delivery modes were significantly associated with an enhancement in PA when compared to the counterparts.

### Limitations

This meta-analysis showed that mHealth interventions could promote PA among the included working populations. However, this study has several limitations that need to be addressed. First, the findings of this study should be interpreted cautiously, considering the relatively small sample sizes and short-term intervention periods (mean 20, SD 14.77 weeks) without follow-up (only 3/8, 38% of the studies reported follow-up). The maintenance of health behavior change is crucial for health promotion practice [40]. Hence, studies with a larger sample size and an extended follow-up period are needed to increase the generalizability of our findings. In addition, including studies with a small sample size may result in errors owing to small-study effects because the effect size might be relatively large. Second, although the heterogeneity was lowered in the subgroup analysis, there was substantial heterogeneity in the main analysis of this study. The possible explanation is that the heterogeneity is because of the additional intervention contents, difference in frequency, intervention duration, and delivery methods. Third, it is considered necessary to compare the differences between PA promotion programs with behavior change and weight management programs. Unfortunately, in the studies included in our analysis, it was difficult to separate them into 2 distinct classifications. Fourth, our search was restricted only to full-text articles published in English or Korean; thus, language and publication bias might have resulted when relevant studies outside the current scope were excluded. Finally, the outcome variables in this meta-analysis were excluded by using only subjective, self-reported data from the previous studies (5/8, 63%). Although the subjective measurement of PA was performed using validated tools (International PA Questionnaire, Short Questionnaire to Assess Health-Enhancing PA, etc), the potential bias for self-reported data cannot be ignored.

### Comparison With Previous Work

Mobile technologies (eg, mobile phones, tablets, and tracking devices) have offered an innovative delivery method for promoting PA in public health practice [41]. Although many scholars have used mHealth interventions as a useful method for behavior change, their effectiveness remains uncertain [35]. Moreover, there is a review deficit for the target populations and settings using mHealth for PA promotion and weight loss. Indeed, there was a literature review that concluded that mHealth interventions for workers are a practical and effective way to promote PA [33]. However, a meta-analysis related to this review [33] was not performed owing to the heterogeneity of the studies’ outcomes and methods and incomplete reporting. To our knowledge, this meta-analysis is the first study to examine the effectiveness of mHealth interventions for PA promotion and weight loss in the working population.

Despite a lack of review studies on the working population, various reviews with the general adult population have shown a positive effect of mHealth in promoting PA. These previous studies concluded that interventions comprising wearable devices and smartphone apps effectively promoted PA in adults, with small to moderate effects (SMD 0.43, 95% CI 0.03-0.82; SMD 0.27, 95% CI 0.15-0.39) [42,43]. Similar conclusions were reported in a meta-regression study [44]. Furthermore, Schoeppe et al [45] found significant PA improvement via smartphone apps. However, there were nonsignificant differences in PA observed by Flores et al [46] (SMD 0.40, 95% CI –0.07 to 0.87), Direito et al [20] (SMD 0.14, 95% CI –0.12 to 0.41), and Islam et al [47] (MD 0.17, 95% CI –2.21 to 2.55).

This study showed evident, positive effects for walking activity using subgroup analysis. The finding agrees with the results of study by Tang et al [48], which reported that the use of a wearable tracker was associated with improvements in PA, especially in the number of steps (SMD 0.332, 95% CI 0.16-0.50). Gal et al [42] and Feter et al [49] also reported that interventions using mobile phones have resulted in significant enhancement on the number of steps (SMD 0.51, 95% CI 0.12-0.91; MD 735, 95% CI 28-1243, respectively). However, Romeo et al [50] and Direito et al [20] could not find significant improvements in walking activity (MD 477, 95% CI –230 to 1183; SMD 0.14, 95% CI –0.01 to 0.29, respectively). In this study, a small effect on overall PA and walking activity was observed. Given the heterogeneity of the included studies, the potential effects of promoting overall PA and walking activity by mHealth interventions cannot be ignored.

Following the recommendation of a previous review, mHealth intervention programs for improving PA should focus on participants’ weight, waist circumference, and BMI [51]. Hence, we considered weight as a secondary outcome with several mHealth intervention studies among the included populations. Islam et al [47] evaluated the effectiveness of mHealth interventions for weight management and found a small but significant loss. In addition, previous studies reported pooled effects of interventions via smartphone app on weight loss (–1.04 kg, 95% CI –1.75 to –0.34; –2.56 kg, 95% CI –3.46 to –1.65) [46,52]. However, the effect of secondary analysis on weight loss was not statistically significant in this study. A previous review revealed that weight management programs combining PA and diet were more effective than interventions with PA alone for weight loss [53]. We hypothesized that one of the reasons for the inconsistent results for weight loss could be the differences in the interventional focus of the study. Indeed, half of the studies included secondary analysis on weight loss (4/8, 50%) and intervention contents for improving PA and diet. Among these 4 studies, 2 (50%) studies reported significant weight loss [28,29]. We can confirm that the studies that obtained significant weight loss results included an intensive focus on diet behavior compared with other studies. A previous study had also emphasized the importance of dietary change in the weight loss program [53]. Moreover, the variability in participants’ characteristics and interventions’ intensity, duration, and type in each study could make the results inconsistent. The inclusion of a study with overweight workers is also a probable cause for the different outcomes. Moreover, there was no clear evidence of benefit from interventions with a wearable tracker for weight loss or PA in overweight populations [48].

In addition, it was impossible to draw any definitive conclusions on the relative effectiveness of different delivery methods owing to considerable heterogeneity and the small number of high-quality studies. However, we found evidence that stand-alone mHealth interventions with no additional *offline* components were less likely to increase PA. The evidence supported the results of various studies and could lead to more robust results. Previous reviews have suggested that behavioral and health outcomes of multicomponent interventions are better than those of stand-alone mHealth interventions [45]. Islam et al [47] also reported that various delivery channels are deemed effective for reducing weight and maintaining BMI. The mHealth technologies incorporated into existing programs by educational support were reported to be beneficial [51].

On the other hand, a previous review has pointed out that mHealth devices were mainly used for outcome measurement or as a supplement to other intervention components [54]. In addition, another review suggested that most mHealth interventions support increasing PA levels, especially by using SMS text messaging and facilitating self-monitoring [55]. In this study, most of the included studies used 2 or more mHealth technologies, and some of them used mHealth devices for outcome measurements. On the basis of the mixed results, a clearer conclusion could be inferred through analysis by including more studies in the future.

### Recommendations for Future Research

This study examined the effectiveness of mHealth intervention for PA improvement among workers and found a modest benefit of the studies. The role of behavior change techniques within the mHealth interventions can also affect PA [44,47]. It was impossible to analyze the effectiveness of each behavior change technique as they were mixed with other intervention characteristics. Hence, further research should include research questions on related aspects for the behavior change techniques. In addition, it was difficult to compare the differences between behavior change programs and weight management programs in this analysis. Finally, further studies to assess feasibility, long-term impact with follow-up, and engagement of mHealth interventions are recommended. This study could not estimate which delivery modes were most likely to change behaviors owing to the few high-quality studies and heterogeneity. With more extensive studies, we propose to analyze the effectiveness of the type of delivery mode on behavior changes in future research.

### Conclusions

Although the overall effects might be relatively small, mHealth interventions appeared to be effective for improving PA among workers. Multicomponent interventions using mHealth devices were more effective than stand-alone uses of mHealth devices. Future studies, including a larger sample size with extended periods, are required to evaluate the effects of behavior change techniques within mHealth interventions on workers’ PA improvement and weight management.

## Appendix

Multimedia Appendix 1 Electronic search strategy in electronic databases.

Multimedia Appendix 2 Program objectives and contents among included studies.

Multimedia Appendix 3 Sensitivity analysis for physical activity.

Multimedia Appendix 4 Sensitivity analysis for weight loss.

## Abbreviations

MD: mean difference
mHealth: mobile health
PA: physical activity
PRISMA: Preferred Reporting Items for Systematic Reviews and Meta-Analyses
RCT: randomized controlled trial
SMD: standardized mean difference
