# Supplementary material for: Promoting Physical Activity and Weight Loss With mHealth Interventions Among Workers: Systematic Review and Meta-analysis of Randomized Controlled Trials
Source: JMIR Mhealth Uhealth. 2022 Jan 21;10(1):e30682. doi: 10.2196/30682 (PMC8817216; doi:10.2196/30682)
Supplement: Multimedia Appendix 1 [file mhealth_v10i1e30682_app1.pdf]

Multimedia Appendix 1. Electronic Search strategy in electronic databases

PubMed
((((((((mhealth) OR (mobile and (program or promotion or intervention))) OR (mhealth and (program or promotion or intervention))) OR (m-health*)) OR (Cell Phone)) OR (smart phone)) OR (Telemedicine)) AND ((((((((quasi-experiment*) OR (experimental study)) OR (Intervention study)) OR (non-RCT)) OR (Evaluation Study)) OR (Controlled Clinical Trial)) OR (Clinical Trial)) OR (Randomized Controlled Trial))) AND ((((((((((((workforce*) OR (Occupational Groups)) OR (worker*)) OR (employee*)) OR ("working adult")) OR ("working population")) OR (workplace*)) OR (worksite*)) OR (Occupational Health Services)) OR (Occupational Health)) OR (Occupational Health Nursing)) OR (employee health promotion))
Results : 2,548 (20.12.08.)

Embase
'mobile health'/exp OR 'mhealth'/exp OR m-health OR (('mobile' OR 'smart') AND phone/exp) OR ‘mobile application*’/exp OR 'mobile health application*' OR 'telehealth*' OR 'text messaging'/exp OR (‘mobile*’ AND (promotion OR program OR management OR intervention)) AND (‘worker*’ OR 'employee*' OR 'workforce'/exp OR 'workplace*’/exp OR ‘worksite*’/exp OR ‘working population’ OR ‘working adult*’ OR ‘occupational health service’/exp OR ‘occupational health*’) AND (‘intervention*’/exp OR 'randomized controlled trial'/exp OR 'controlled study'/exp OR 'pretest posttest design'/exp OR 'experimental study'/exp OR ‘quasi experimental study’/exp OR 'evaluation study'/exp OR ‘program evaluation’ OR ‘program effectiveness’)
Results : 2,148 (20.12.09.)

CINAHL complete
((((((((mhealth) OR (mobile and (program or promotion or intervention))) OR (mhealth and (program or promotion or intervention))) OR (mhealth*)) OR (Cell Phone)) OR (smart phone)) OR (Telemedicine)) AND ((((((((quasi-experiment*) OR (experimental study)) OR (Intervention study)) OR (non-RCT)) OR (Evaluation Study)) OR (Controlled Clinical Trial)) OR (Clinical Trial)) OR (Randomized Controlled Trial))) AND ((((((((((((workforce*) OR (Occupational Groups)) OR (worker*)) OR (employee*)) OR ("working adult")) OR ("working population")) OR (workplace*)) OR (worksite*)) OR (Occupational Health Services)) OR (Occupational Health)) OR (Occupational Health Nursing)) OR (employee health promotion))
Results : 230 (20. 12. 08.)

Cochrane Library
((((((((mhealth) OR (mobile and (program or promotion or intervention))) OR (mhealth and (program or promotion or intervention))) OR (m-health*)) OR (Cell Phone)) OR (smart phone)) OR (Telemedicine)) AND ((((((((quasi-experiment*) OR (experimental study)) OR (Intervention study)) OR (non-RCT)) OR (Evaluation Study)) OR (Controlled Clinical Trial)) OR (Clinical Trial)) OR (Randomized Controlled Trial))) AND ((((((((((((workforce*) OR (Occupational Groups)) OR (worker*)) OR (employee*)) OR ("working adult")) OR ("working population")) OR (workplace*)) OR (worksite*)) OR (Occupational Health Services)) OR (Occupational Health)) OR (Occupational Health Nursing)) OR (employee health promotion))
Results : 29 cochrane reviews + 1,300 Trials (20. 12. 09.)
