# Supplementary material for: Promoting Physical Activity and Weight Loss With mHealth Interventions Among Workers: Systematic Review and Meta-analysis of Randomized Controlled Trials
Source: JMIR Mhealth Uhealth. 2022 Jan 21;10(1):e30682. doi: 10.2196/30682 (PMC8817216; doi:10.2196/30682)
Supplement: Multimedia Appendix 2 [file mhealth_v10i1e30682_app2.pdf]

Multimedia Appendix 2. Program objectives and contents among included studies

Study	Program objectives	Program contents	
		Physical Activity	Diet
Van Wier et al [28]	weight management + behavioral change	Y	Y
Kim et al [25]	weight management + behavioral change	Y	Y
Júdice et al [23]	behavioral change	Y	N
Kim et al [24]	weight management	Y	Y
Brakenridge et al [22]	behavioral change	Y	N
Simons et al [27]	behavioral change	Y	N
Viester et al [29]	weight management + behavioral change	Y	Y
Rollo and Prapavessis [26]	behavioral change	Y	N
