# Supplementary material for: Promoting Physical Activity and Weight Loss With mHealth Interventions Among Workers: Systematic Review and Meta-analysis of Randomized Controlled Trials
Source: JMIR Mhealth Uhealth. 2022 Jan 21;10(1):e30682. doi: 10.2196/30682 (PMC8817216; doi:10.2196/30682)

Multimedia Appendix 3. Sensitivity analysis: physical activity

Study omitted	Estimate	95% CI
1	0.081	0.000-0.162
2	0.193	0.098-0.288
3	0.095	0.022-0.168
4	0.094	0.021-0.167
5	0.095	0.019-0.170
6	0.089	0.014-0.164
7	0.088	0.012-0.163
8	0.120	0.045-0.195
9	0.107	0.032-0.181
10	0.110	0.035-0.185
11	0.096	0.020-0.173
12	0.095	0.021-0.169
Combined	0.103	0.029-0.176

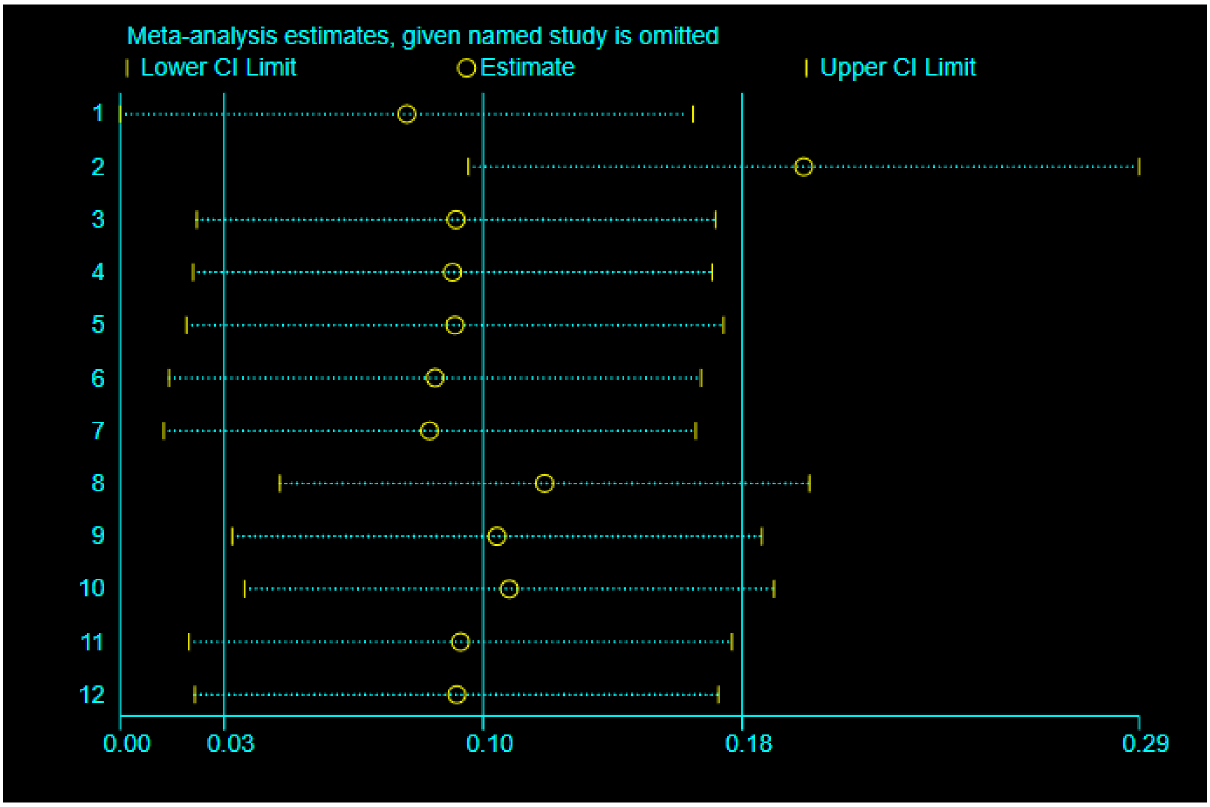

Supplement: Multimedia Appendix 3 [file mhealth_v10i1e30682_app3.pdf]
