# Supplementary figures and images for: Promoting Physical Activity and Weight Loss With mHealth Interventions Among Workers: Systematic Review and Meta-analysis of Randomized Controlled Trials
Source: JMIR Mhealth Uhealth. 2022 Jan 21;10(1):e30682. doi: 10.2196/30682 (PMC8817216; doi:10.2196/30682)

#### Multimedia Appendix 4. Sensitivity analysis: weight loss

| Study omitted | Estimate | 95% CI       |
|---------------|----------|--------------|
| 1             | 0.031    | -0.068-0.130 |
| 2             | -0.043   | -0.160-0.075 |
| 3             | 0.023    | -0.064-0.110 |
| 4             | 0.035    | -0.053-0.123 |
| Combined      | 0.017    | -0.066-0.101 |

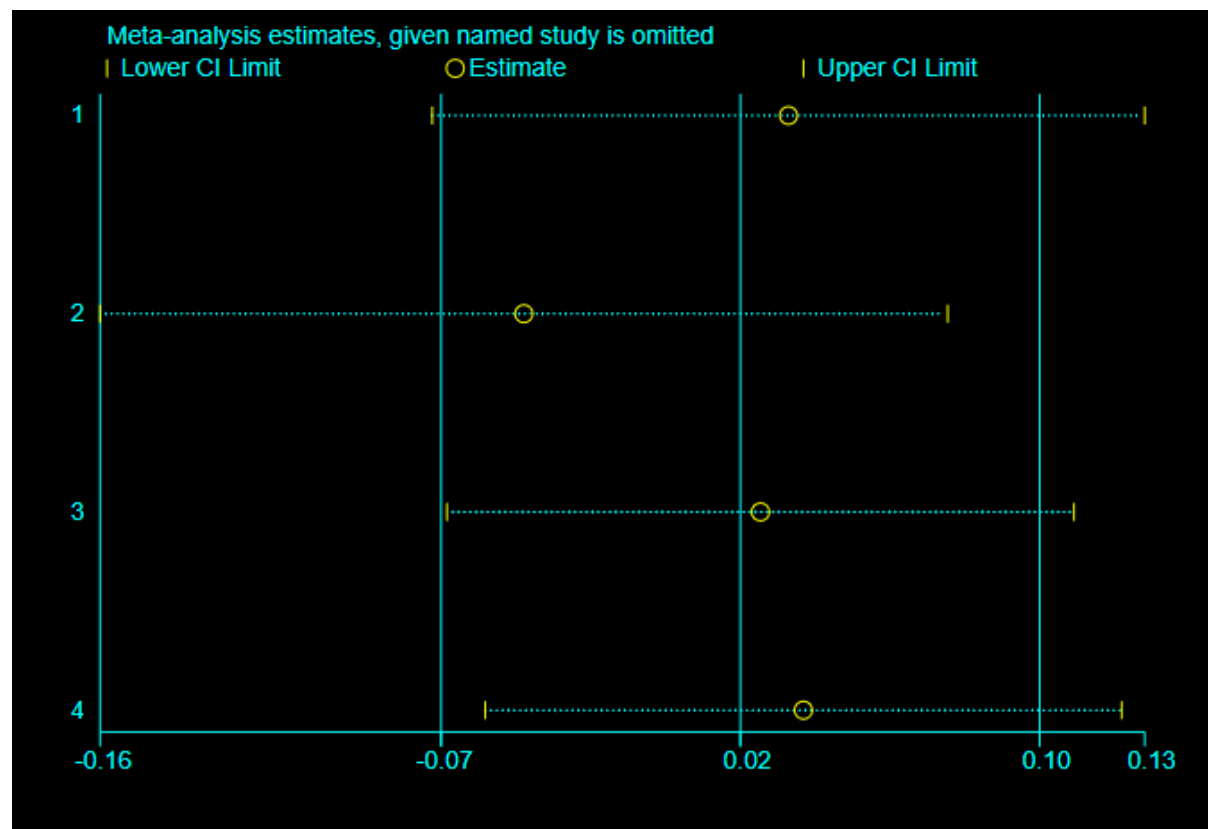

Supplement: Multimedia Appendix 4 [file mhealth_v10i1e30682_app4.pdf]
